# Mobocertinib in Patients with EGFR Exon 20 Insertion-Positive Non-Small Cell Lung Cancer (MOON): An International Real-World Safety and Efficacy Analysis

**DOI:** 10.3390/ijms25073992

**Published:** 2024-04-03

**Authors:** Oliver Illini, Felix Carl Saalfeld, Petros Christopoulos, Michaël Duruisseaux, Anders Vikström, Nir Peled, Ingel Demedts, Elizabeth Dudnik, Anna Eisert, Sayed M. S. Hashemi, Urska Janzic, Waleed Kian, Katja Mohorcic, Saara Mohammed, Maria Silvoniemi, Sacha I. Rothschild, Christian Schulz, Claas Wesseler, Alfredo Addeo, Karin Armster, Malinda Itchins, Marija Ivanović, Diego Kauffmann-Guerrero, Jussi Koivunen, Jonas Kuon, Nick Pavlakis, Berber Piet, Martin Sebastian, Janna-Lisa Velthaus-Rusik, Luciano Wannesson, Marcel Wiesweg, Robert Wurm, Corinna Albers-Leischner, Daniela E. Aust, Melanie Janning, Hannah Fabikan, Sylvia Herold, Anna Klimova, Sonja Loges, Yana Sharapova, Maret Schütz, Christoph Weinlinger, Arschang Valipour, Tobias Raphael Overbeck, Frank Griesinger, Marko Jakopovic, Maximilian J. Hochmair, Martin Wermke

**Affiliations:** 1 Department of Respiratory and Critical Care Medicine, Klinik Floridsdorf, Vienna Healthcare Group, Bruenner Straße 68, A-1210 Vienna, Austriamaximilian.hochmair@gesundheitsverbund.at (M.J.H.); 2 Karl Landsteiner Institute of Lung Research and Pulmonary Oncology, A-1210 Vienna, Austria; 3 Clinic for Internal Medicine I, University Hospital Carl Gustav Carus, TU Dresden, 01307 Dresden, Germany; felix.saalfeld@ukdd.de (F.C.S.); martin.wermke@ukdd.de (M.W.); 4 National Center for Tumor Diseases, 01307 Dresden, Germany; 5 National Network Genomic Medicine Lung Cancer (nNGM), 50937 Cologne, Germany; 6 Thoraxklinik and Translational Lung Research Center (TLRC), member of the German Center for Lung Research (DZL), Heidelberg University Hospital, 69126 Heidelberg, Germany; 7 Respiratory Department and Early Phase, Louis Pradel Hospital, Hospices Civils de Lyon Cancer Institute, 69002 Lyon, France; 8 Oncopharmacology Laboratory, Cancer Research Center of Lyon, Unité Mixte de Recherche (UMR), Institut National de la Santé et de la Recherche Médicale (INSERM), 1052 Centre National de la Recherche Scientifique (CNRS), 5286 Lyon, France; 9 Université Claude Bernard, Université de Lyon, 69622 Villeurbanne cedex, France; 10 Department of Pulmonary Medicine, University Hospital Linköping, 58185 Linköping, Sweden; 11 The Hemsely Cancer Center, Shaare Zedek Medical Center, Jerusalem 9103102, Israel; 12 Department of Pulmonary Diseases, AZ Delta, Deltalaan 1, 8800 Roeselare, Belgium; ingel.demedts@azdelta.be; 13 Head, Thoracic Oncology Service, Assuta Medical Centers, Tel-Aviv 6329302, Israel; 14 Faculty of Health Sciences, Ben-Gurion Unversity of the Negev, Be’er Sheva 84105, Israel; 15 Lung Cancer Group Cologne, Department I for Internal Medicine and Center for Integrated Oncology Aachen Bonn Cologne Dusseldorf, Faculty of Medicine and University Hospital of Cologne, University of Cologne, 50937 Cologne, Germany; 16 Department of Pulmonary Medicine, Amsterdam UMC, VU University Medical Center, Cancer Center Amsterdam, 1081 Amsterdam, The Netherlands; 17 Medical Faculty, University of Ljubljana, 1000 Ljubljana, Slovenia; urska.janzic@klinika-golnik.si (U.J.);; 18 Medical Oncology Unit, University Clinic Golnik, 4204 Golnik, Slovenia; 19 Institute of Oncology, Assuta Ashdod University Hospital, Ashdod 7747629, Israel; 20 Kent Oncology Centre, Maidstone and Tunbridge Wells NHS Trust, Kent TN24QJ, UK; 21 Department of Pulmonary Diseases, Turku University Hospital, University of Turku, 20014 Turku, Finland; 22 Center for Oncology & Hematology and Comprehensive Cancer Center, Cantonal Hospital Baden, 5404 Baden, Switzerland; 23 Department of Internal Medicine II, University Hospital, 93053 Regensburg, Germany; 24 Department of Pneumology, Asklepios Tumorzentrum Hamburg, Klinikum Harburg, 21075 Hamburg, Germany; 25 Oncology Department, University Hospital Geneva, 1205 Geneva, Switzerland; 26 Department of Pneumology, Universitätsklinikum Krems, 3500 Krems an der Donau, Austria; 27 Department of Medical Oncology, Royal North Shore Hospital, St Leonards, NSW 2065, Australia; 28 Northern Clinical School, University of Sydney, St Leonards, NSW 2065, Australia; 29 Department of Oncology, University Medical Centre Maribor, 2000 Maribor, Slovenia; 30 Division of Respiratory Medicine and Thoracic Oncology, Department of Medicine V, Thoracic Oncology Center Munich, University Hospital, University of Munich (LMU), 81377 Munich, Germany; 31 Department of Oncology and Radiotherapy, Oulu University Hospital, 90014 Oulu, Finland; 32 Cancer and Translational Medicine Research Unit, University of Oulu, 90014 Oulu, Finland; 33 Medical Research Center Oulu, 90014 Oulu, Finland; 34 Department Thoracic Oncology, SLK Fachklinik Löwenstein, 74245 Löwenstein, Germany; 35 Department of Respiratory Medicine, Radboudumc, 6225 GA Nijmegen, The Netherlands; 36 Department of Medicine, Hematology/Oncology, University Hospital, University of Frankfurt, 60596 Frankfurt am Main, Germany; 37 Department of Oncology, Hematology and Bone Marrow Transplantation with Section Pneumology, Hubertus Wald Comprehensive Cancer Center Hamburg, University Medical Center Hamburg-Eppendorf, 20251 Hamburg, Germany; 38 Istituto Oncologico della Svizzera Italiana, 6500 Bellinzona, Switzerland; 39 West German Cancer Center, Department of Medical Oncology, University Duisburg-Essen, 45147 Essen, Germany; 40 Division of Pulmonology, Department of Internal Medicine, LKH-Universitätsklinikum, Medical University of Graz, 8036 Graz, Austria; 41 Department for Pathology, University Hospital Carl Gustav Carus, TU Dresden, 01307 Dresden, Germany; 42 DKFZ-Hector Cancer Institute at the University Medical Center Mannheim, 68167 Mannheim, Germany; 43 Department of Personalized Oncology, University Hospital Mannheim, Heidelberg University, 68167 Mannheim, Germany; 44 Division of Personalized Medical Oncology, German Cancer Research Center (DKFZ), 69120 Heidelberg, Germany; 45 German Center for Lung Research (DZL), 69120 Heidelberg, Germany; 46 Core Unit for Data Management and Analytics, National Center for Tumor Diseases, 01307 Dresden, Germany; 47 Department of Hematology and Medical Oncology, University Medical Center Göttingen, Göttingen University, 37075 Göttingen, Germany; 48 Department of Hematology and Oncology, Pius University Hospital, University Medicine Oldenburg, 26121 Oldenburg, Germany; 49 Department for Respiratory Diseases Jordanovac, University Hospital Center Zagreb, 10000 Zagreb, Croatia; 50 School of Medicine, University of Zagreb, 10000 Zagreb, Croatia

**Keywords:** non-small cell lung cancer, EGFR exon 20 inhibitors, mobocertinib, real-world data, exon 20 insertion

## Abstract

EGFR exon 20 (EGFR Ex20) insertion mutations in non-small cell lung cancer (NSCLC) are insensitive to traditional EGFR tyrosine kinase inhibitors (TKIs). Mobocertinib is the only approved TKI specifically designed to target EGFR Ex20. We performed an international, real-world safety and efficacy analysis on patients with EGFR Ex20-positive NSCLC enrolled in a mobocertinib early access program. We explored the mechanisms of resistance by analyzing postprogression biopsies, as well as cross-resistance to amivantamab. Data from 86 patients with a median age of 67 years and a median of two prior lines of treatment were analyzed. Treatment-related adverse events (TRAEs) occurred in 95% of patients. Grade ≥3 TRAEs were reported in 38% of patients and included diarrhea (22%) and rash (8%). In 17% of patients, therapy was permanently discontinued, and two patients died due to TRAEs. Women were seven times more likely to discontinue treatment than men. In the overall cohort, the objective response rate to mobocertinib was 34% (95% CI, 24–45). The response rate in treatment-naïve patients was 27% (95% CI, 8–58). The median progression-free and overall survival was 5 months (95% CI, 3.5–6.5) and 12 months (95% CI, 6.8–17.2), respectively. The intracranial response rate was limited (13%), and one-third of disease progression cases involved the brain. Mobocertinib also showed antitumor activity following EGFR Ex20-specific therapy and vice versa. Potential mechanisms of resistance to mobocertinib included amplifications in MET, PIK3CA, and NRAS. Mobocertinib demonstrated meaningful efficacy in a real-world setting but was associated with considerable gastrointestinal and cutaneous toxicity.

## 1. Introduction

Mutations in the EGFR tyrosine kinase domain are drivers of the development of non-small cell lung cancer (NSCLC). Patients with EGFR exon 20 (EGFR Ex20) insertions represent an uncommon subset of approximately 5–12% of EGFR-mutant NSCLC patients [1,2]. Advanced NSCLC with EGFR Ex20 mutations is difficult to treat and is associated with a poor prognosis [3]. Due to primary resistance to conventional EGFR tyrosine kinase inhibitors (TKIs) and immunotherapy [4,5,6,7], the standard first-line therapy until recently (March 2024) was platinum-doublet chemotherapy with or without immune checkpoint inhibitors [4]. The addition of the anti-MET/anti-EGFR bispecific antibody amivantamab to platinum-doublet chemotherapy has significantly improved outcomes and thus defined the new standard of care, albeit at the cost of increased toxicity. 

Mobocertinib is the only approved TKI with specific activity against EGFR Ex20 mutation-positive NSCLC. Since its approval was based on an expansion cohort from a phase I/II, single-arm trial in patients pretreated with platinum but not necessarily amivantamab, important questions remain to be answered. These include transferability of the clinical trial observations to the real-world setting, activity following amivantamab pretreatment, and mechanisms of resistance. In addition, the efficacy in previously untreated patients as well as in patients with brain metastases is currently unknown.

In this study, we report our experience with a large cohort of EGFR Ex20 mutation-positive patients treated with mobocertinib in an early access program, which significantly extends the data from the prospective EXCLAIM trial [8]. We provide the first assessment of the tolerability and efficacy of mobocertinib in a large number of both pretreated and treatment-naïve real-world patients. Finally, we explore potential mechanisms of resistance to mobocertinib and cross-resistance to amivantamab.

## 2. Results

### 2.1. Patients

Data from 86 patients with EGFR Ex20 mutation-positive NSCLC treated with mobocertinib in an early access program were included in this analysis. Clinical and pathologic characteristics are presented in Table 1 and Supplement Table S1. The median age was 67 years (range, 25–88), 69% were women, 33% had brain metastases at baseline, and 9% presented with an unfavorable performance status (ECOG PS ≥ 2). Most patients (63%) were never smokers.

With a median age of 75 years, the fourteen treatment-naïve patients (16%) who received mobocertinib as the first line of palliative therapy were older than the pretreated patients (median age, 65 years). Pretreated patients (n = 72; 84%) had received a median of two previous lines of therapies (range, 1–8), including platinum-doublet +/− immunotherapy in most cases (81%). Prior to treatment with mobocertinib, one out of four (25%) pretreated patients received at least one EGFR inhibitor, and 12% had already been treated with a drug specifically targeting EGFR Ex20 (amivantamab or poziotinib).

Except for two patients with adenosquamous carcinoma, the predominant histologic subtype was adenocarcinoma (98%). A TP53 commutation was present in 26% of patients. The EGFR Ex20 subtype was near-loop in 80%, far-loop in 15%, and of another classification in 5% of cases. For detailed description of EGFR mutation subtypes see Supplement Table S4.

### 2.2. Efficacy

#### 2.2.1. Response

**Figure 1:**
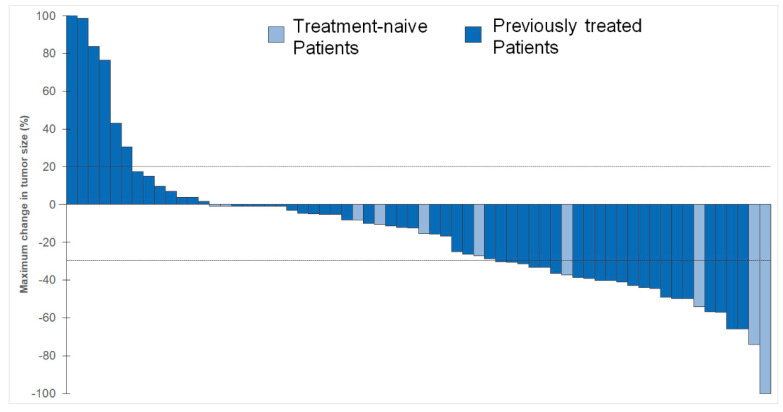
Response of target lesions in treatment-naïve and pretreated patients. Best response to mobocertinib. Waterfall plot of maximum change in tumor size on imaging measured according to RECIST v1.1 in all target lesions between baseline and follow-up in pretreated and treatment-naïve patients. Both growth (+20%) and shrinkage (−30%) of tumors are indicated by the dashed lines. One patient experienced a tumor growth of 400%. For better illustration purposes, the Y-axis only shows 100%. Patients with no shrinkage or growth are shown with −1%. Eighteen patients are not shown because there was no measurable disease or because they had no adequate response for assessment. Four patients are not shown because retrospective measurement of lesions was not possible.

The results for efficacy parameters are presented in Table 2. An objective response to mobocertinib was observed in 33.7% (95% CI, 23.9–44.7) of patients, with 1 patient (1.2%) achieving a CR and 28 patients (32.6%) a PR. Nonresponders consisted of 11 patients (12.8%) with PD, 33 patients (38.4%) with SD, and 13 patients (15.1%) in whom the response was not evaluable. The maximum change in tumor size relative to baseline in 65 patients is presented in Figure 1.

In the 29 patients achieving a tumor response, the median DOR was 8 months (95% CI, 3.7–12.3). At the data cutoff, 38% of responses were still ongoing. The ORR was similar in treatment-naïve and pretreated patients (34.7% versus 28.7%).

#### 2.2.2. Progression-Free Survival and Overall Survival

**Figure 2:**
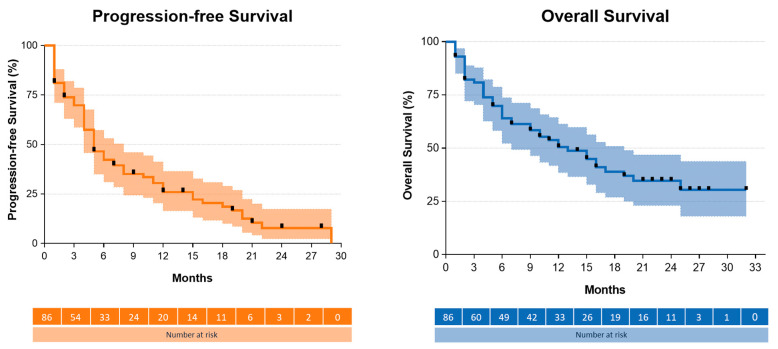
Progression-free and overall survival from the start of mobocertinib treatment. Kaplan–Meier plots with 95% confidence intervals. Black rectangles mark censoring. The median PFS was 5 months (95% CI, 3.5–6.5). The median OS was 12 months (95% CI, 6.8–17.2).

At the data cut-off, 55% of patients had died. After a median follow-up of 21 months (95% CI, 17.7–24.3), the median OS was 12 months (95% CI, 6.8–17.2) in the entire group of patients (Figure 2). In pretreated patients, the median OS reached 12 months (95% CI, 7.1–16.9), whereas the OS data were not mature in treatment-naïve patients.

The median PFS was 5 months (95% CI, 3.5–6.5). At the data cutoff, 80% of patients had disease progression. Using Cox regression, brain metastases at baseline (3 months versus 8 months, HR = 0.358 (0.209–0.615), *p* < 0.001) and discontinuation of mobocertinib therapy because of TRAEs (4 months versus 8 months, HR = 0.426 (0.231–0.787), *p* = 0.006) were significantly associated with shorter PFS. Moreover, a poor ECOG performance status showed a nonsignificant trend for predicting shorter PFS (ECOG PS 0 versus ECOG PS ≥ 1, 4 months versus 7 months, HR = 0.585 (0.358–0.956, *p* = 0.32). Age, sex, line of therapy, the presence of a TP53 commutation, and EGFR mutational subgroup (near-loop versus far-loop) were not associated with PFS in our analysis (Table S2).

#### 2.2.3. Intracranial Outcome

New or progressive brain metastases were found in 15 patients at the time of starting mobocertinib. Seven of those patients had not received cranial irradiation during or immediately before mobocertinib therapy and had measurable brain metastasis. Of those patients, one showed an intracranial (ic) PR (14.3%), two patients showed icSD (28.6%), and one patient showed icPD (14.3%); three patients were not evaluable for response assessment or had no measurable disease.

In 18 (32%) out of 56 patients who experienced disease progression and were evaluable, the disease progression involved the central nervous system.

#### 2.2.4. Sequencing EGFR Ex20-Targeted Therapy

Ten patients had received EGFR Ex20-specific therapy (amivantamab +/− lazertinib or poziotinib) prior to the initiation of mobocertinib. In this group, the best response to mobocertinib was a PR in three patients (30%), SD in 5 patients (50%), and PD or NE in one patient each (each 10%).

Twelve patients received amivantamab following progression on mobocertinib (Figure S1). The median duration of amivantamab treatment in these patients was 6 months (95% CI, 3.4–8.6). At the time of the data cutoff, six patients were still receiving therapy. Two patients (17%) showed a PR, and eight patients (67%) showed SD as the best response to amivantamab. The tumor response of two patients was not evaluable.

### 2.3. Safety

Most patients (95%) experienced TRAEs of any grade (Table 3), the most common being diarrhea (77%), rash (59%), and dry skin (40%). TRAEs reported in at least 20% of patients are graphically presented in Figure S2. Grade ≥ 3 TRAEs were reported in 38% of patients, and diarrhea (22%) was the only grade ≥3 TRAE that occurred in more than 10% of patients. One patient died due to diarrhea (grade 5) in combination with grade 4 vomiting, which led to renal failure and death despite hospitalization and the application of intravenous fluids. Another patient died due to liver toxicity (grade 5) in combination with disseminated intravascular coagulopathy, although the progression of liver and brain metastases may have contributed to this fatal event.

The majority of patients (83%/n = 71) started with a standard mobocertinib dose of 160 mg QD. The remaining patients were started with lower doses, as per local investigator decision, in order to reduce the risk of severe adverse events. In those 15 patients, the median starting dose was 120 mg QD, and the dose was increased by one dose level (40 mg) in 7 cases (47%). Among all patients, dose reduction and treatment interruption due to the occurrence of TRAEs was reported in 51% and 42%, respectively. Treatment was permanently discontinued in 17% of patients because of TRAEs, including diarrhea (13%), vomiting (6%), nausea (5%), and mucositis (3%).

Women were more likely to discontinue treatment due to TRAEs than men (27.1% of female versus 3.7% of male patients). This difference was statistically significant (*p* = 0.025). Dose reductions (63% versus 26%) and interruptions (53% versus 19%) were also more common in women. By direct comparison, gastrointestinal TRAEs were reported more frequently in women (diarrhea 83%, nausea 42%, anorexia 37%, and vomiting 25% versus 63%, 19%, 26%, and 15% in men, respectively), while men were slightly more affected by cutaneous toxicity (rash 81%, dry skin 56%, paronychia 37%, and pruritus 30% versus 49%, 32%, 29%, and 22% in women, respectively). Age and ECOG performance status were not associated with the discontinuation of treatment due to TRAEs.

### 2.4. Mechanisms of Resistance

#### 2.4.1. Analysis of Posttreatment Biopsies

**Figure 3:**
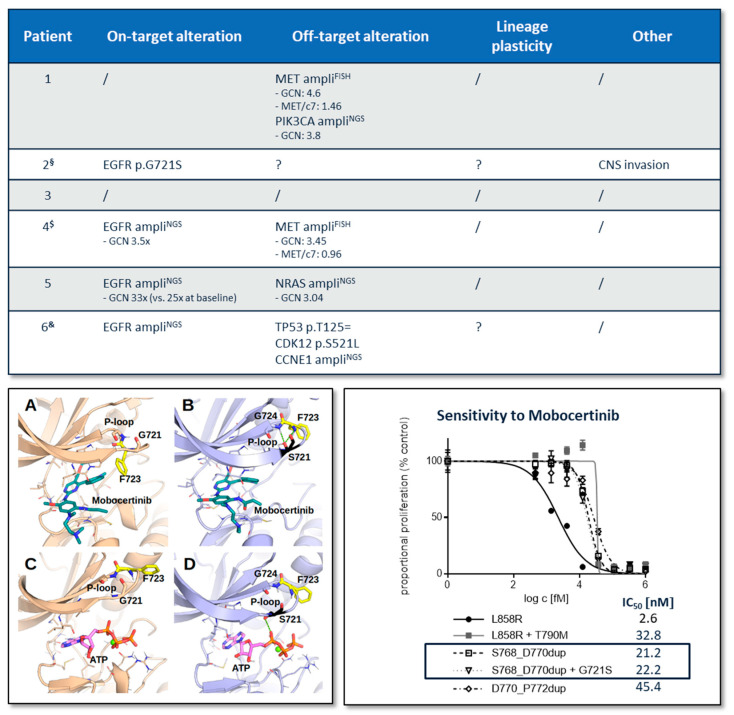
Mechanisms of resistance to mobocertinib. *Upper panel*: Potential mechanisms of resistance that have been detected in post- but not in pretreatment tissue biopsies. Paired samples were assessed by next-generation sequencing (NGS) using custom DNA and RNA panels developed for recurrent genetic alterations in lung cancer, by fluorescence in situ hybridization (FISH) for MET and HER2 amplification, and by a board-certified pathologist to identify transformation to small or squamous cell histology (lineage plasticity). Ampli: Amplification (superscript indicates whether it was detected by NGS or FISH). GCN: Gene copy number. MET/c7: Ratio of MET signals to centromere 7 signals in FISH. §: Posttreatment sample was a cerebrospinal fluid cytology specimen. Therefore, only a small NGS panel analysis could be performed. $: Pretreatment biopsy could only be analyzed for hotspots in BRAF, EGFR, and KRAS (Sanger sequencing). &: Both samples were liquid biopsies analyzed for plasma-circulating tumor DNA. *Lower left panel*: 3D-models of EGFR TK complexes with TKI mobocertinib (teal) and its competitive substrate ATP (magenta) obtained from MD: (**A**) p.768_770dup with mobocertinib: a flexible P-loop assumes a conformation when its F723 forms a hydrophobic contact with mobocertinib, shielding it from solvent; (**B**) p.768_770dup + p.G721S with mobocertinib: S721 forms hydrogen bond(s) with F723 and G724, rigidifying the P-loop and shifting F723 away from the binding pocket, exposing mobocertinib to the solvent; (**C**) p.768_770dup with ATP: the P-loop does not interact with ATP; (**D**) p.768_770dup + G721S with ATP: S721 forms an additional hydrogen bond with oxygens in ATP, either directly or mediated by water molecules. Key amino acid residues interacting with the ligands are shown as thin sticks. A magnesium ion in complexes with ATP is shown as a green sphere. Key hydrogen bonds formed by the Ser721 (black) are shown with green dashes. Non-polar hydrogen atoms and solvent molecules are hidden for better visibility. Images were created with PyMOL. *Lower right panel*: Analysis of the EGFR p.G721S mutation in the Ba/F3 cell model. Cells retrovirally transduced with overexpression vectors encoding the EGFR exon 20 insertion p.S768_D770dup alone or in combination with the acquired EGFR p.G721S variant; controls were deprived of IL-3 and treated with increasing concentrations of mobocertinib. Proportional proliferation compared to the DMSO control and half maximal inhibitory concentration (IC_50_) for mobocertinib are provided.

In six patients, biomaterial obtained following progression on mobocertinib was available, which was tumor tissue in five cases and plasma-derived tumor DNA in one. The specimens were subjected to DNA/RNA panel sequencing and FISH, focusing on recurrent genetic mechanisms of resistance to targeted therapies described in NSCLC. The tissue biopsies were also assessed for morphologic transformation. Emerging genetic alterations compared to pretreatment specimens are summarized in Figure 3. Most importantly, MET, NRAS, PIK3CA, and EGFR amplifications occurred, as well as a previously undescribed variant in EGFR (p.G721S). Morphologic transformation was not detected in any patient.

#### 2.4.2. Characterization of the Emerging EGFR p.G721S Variant

To characterize the potential on-target resistance variant EGFR p.G721S, we carried out computational modeling of the preexisting EGFR p.S768_D770dup mutation with and without the secondary p.G721S variant. Molecular dynamics simulations were used to study the time-dependent behavior of the protein in complex with mobocertinib and ATP, respectively. Structural analysis and quantitative assessment of mutation-induced structural changes and interactions between EGFR and the ligands were used to propose a mechanistic explanation of the acquired mobocertinib resistance. It was observed that the p.G721S mutation induced structural changes in the EGFR active site, which had no effect on mobocertinib affinity (Figure 3, lower left panel: A + B), as the estimated free energies of mobocertinib binding to p.S768_D770dup and p.S768_D770dup + p.G721S were not significantly different (−52.5 ± 1.1 kcal/mol and −52.9 ± 1.5 kcal/mol, respectively). Visual inspection of the obtained data indicated that molecular dynamics frames featuring structural changes induced by the p.G721S mutation (Figure 3, lower left panel: C + D) were characterized by improved affinities to ATP (−27.6 ± 3.5 kcal/mol and −35.1 ± 3.0 kcal/mol, respectively). In principle, this could lead to drug resistance by competitive substrate interference. However, such frames were only scarcely present (i.e., such mutation-induced changes to the binding site were rarely observed over the simulation time), so that the average binding free energies calculated over the entire conformational ensemble characteristic of real-life behavior were not significantly different (−31.0 ± 1.4 kcal/mol and −30.4 ± 5.6 kcal/mol, respectively). In summary, computational modeling did not suggest a significant effect of the acquired p.G721S mutation on the interaction of mobocertinib with the EGFR Ex20 protein.

These results were corroborated in a Ba/F3 EGFR overexpression model (Figure 3, lower right panel). In a proliferation assay, the presence of the p.G721S mutation did not change the IC_50_ for mobocertinib (or to other inhibitors, see Table S3) in cells harboring EGFR p.S768_D770dup (22.2 nM versus 21.2 nM). The variant was detected in a cytology specimen from cerebrospinal fluid in a patient who had developed meningeosis carcinomatosa during mobocertinib treatment. Hence, invasion of the central nervous system might be an alternative mechanism of resistance in this particular patient.

## 3. Discussion

The MOON study is the largest published analysis of a multicenter retrospective dataset focusing on the safety and efficacy of mobocertinib in treatment-naïve and pretreated patients with advanced EGFR Ex20 mutation-positive NSCLC. In comparison to the EXCLAIM study—a phase 1/2 open-label nonrandomized trial [8]—our study confirms the efficacy and challenging safety profile of mobocertinib in a real-world setting.

### 3.1. Efficacy

In our study, mobocertinib demonstrated substantial antitumor activity. The observed ORR of 34% is similar to the reported ORR in the selected EXCLAIM population of 28%. In contrast, both PFS and OS were numerically shorter in the MOON study than in the EXCLAIM study (PFS, 5 versus 7 months; OS, 12 versus 24 months, respectively). Notably, the real-world population in the MOON study presented with less favorable baseline characteristics. In contrast to the EXCLAIM study, patients with a poor performance status (9% ECOG PS ≥ 2) and active brain metastasis (17.4%) were included. In addition, our population had received more lines of prior therapies (median 2 versus 1), and the median age was higher (67 versus 60 years). The presence of brain metastases (significantly) and a poor performance status (trend) were associated with reduced PFS in the MOON study. We therefore believe that the shorter survival compared to that in the EXCLAIM study is in line with what can be expected in a less selected, heavily pretreated real-world population.

### 3.2. Treatment-Naïve Patients

For the first time, we demonstrate that treatment with mobocertinib is both feasible and effective in previously untreated patients who refused chemotherapy or were deemed ineligible by their treating physician. For this older patient population (median age, 75 versus 65 years in the pretreated cohort; range, 50–88 years), mobocertinib might be a valuable first-line treatment option considering that PFS was not different in older versus younger patients (Table S2) and that age was not associated with a higher discontinuation rate.

Whether this applies to previously untreated chemotherapy-eligible patients as well is questionable, as recent preliminary communication suggests that mobocertinib failed to provide significant benefit in comparison to chemotherapy in this setting (EXCLAIM-2) [10]. Of note, the addition of amivantamab to standard first-line chemotherapy was associated with significantly prolonged PFS in the phase III randomized PAPILLON trial and is now considered the new standard-of-care for chemotherapy-eligible patients [11].

### 3.3. Intracranial Activity

In line with previous reports, in the MOON study, approximately one-third of patients with EGFR Ex20 mutations presented with brain metastases at baseline (33%) [3,8]. While current EGFR TKIs for the standard treatment of canonical EGFR mutations have excellent intracranial efficacy, data for mobocertinib are non-existent. In the EXCLAIM study, patients with active brain metastases were excluded [8]. In the MOON study, one of seven nonirradiated patients experienced a partial intracranial tumor response (icORR of 14%). In addition, the central nervous system was involved in one-third of disease progression cases occurring in the MOON study. Considering that the brain was the primary site of disease progression in the EXCLAIM study, along with additional real-world evidence showing an icORR of 0% (n = 8) [12], the intracranial activity of mobocertinib may be very limited. Novel EGFR Ex20-targeting compounds optimized for CNS penetration, such as zipalertinib, might elicit better intracranial responses but may also present challenging toxicity profiles [13].

### 3.4. Safety and Tolerability

The toxicity of mobocertinib remains a concern, as half of the patients (49%) experienced serious adverse events and nearly one in five patients (17%) discontinued treatment due to TRAEs in the EXCLAIM study. In the MOON study, this toxicity profile was confirmed. Nearly all patients (95%) experienced any TRAE, and 38% experienced a grade ≥3 event. In half of our patients (51% versus 25% in the EXCLAIM study), dose reduction due to the emergence of TRAEs was reported, and the treatment was permanently discontinued in 17% of patients. Whereas there were no toxicity-related deaths in the EXCLAIM study, two events in our study were classified as death due to the toxicity of mobocertinib. The incidence of TRAEs of grade ≥3 was similar (38% versus 35%) in patients who had received immunotherapy (n = 34) as the last treatment prior to mobocertinib compared to other therapies.

Interestingly, in the MOON study, women were significantly more likely to discontinue treatment because of TRAEs and to experience gastrointestinal events than men. Other reports shedding light on the sex-specific incidence of TRAEs of EGFR inhibitors are limited. A simple but possible explanation could be that the lower body weight of women could result in a higher relative dose. For the EGFR TKI afatinib, there is evidence that a reduced starting dose for female patients or patients with a low body weight may result in better tolerability, including a reduction in severe diarrhea and rash, without a negative impact on efficacy [14,15]. It has also been shown that the blood concentration of the drug correlates with the severity of diarrhea [16] and that the steady-state plasma concentration was influenced by the oral dose administered [17]. Given this explanation, the risk of severe adverse events could be managed with an initial dose reduction in patients with a lower body weight, in women, or in those with other risk factors. Beyond that, we propose that sex-dependent characteristics of the microbiome may have an impact on the metabolism of mobocertinib and its side effects [18]. The importance of fecal microbiota to TKI tolerability has been demonstrated in a randomized trial showing that fecal microbiota transplantation from healthy donors was effective in alleviating TKI-induced diarrhea in patients with renal cell carcinoma treated with sunitinib or pazopanib [19]. Unfortunately, relevant information, including body weight, characteristics of the gut microbiota, comedication, and sociostructural factors were not available in the MOON study. Additionally, although patients had to present with adequate renal and liver function to participate in the early access program, there was no standardized protocol for monitoring organ function during treatment. It would be valuable if future trials of EGFR inhibitors would report on sex-related differences in relation to toxicity and dosing.

### 3.5. Sequencing EGFR Ex20-Specific Therapies

Both mobocertinib and amivantamab are EGFR Ex20-specific therapies that are available following platinum-based chemotherapy in many countries. In the MOON study, we reported a number of patients who had clinical benefit from amivantamab following mobocertinib treatment and vice versa. Indeed, given the different modes of action of EGFR TKIs and the bispecific anti-MET and anti-EGFR antibody, it is possible that cross-resistance does not occur on a broader scale. Unfortunately, resistance mechanisms to both drugs are still largely unknown. Here, we found that off-target resistance occurs following mobocertinib treatment, which is comparable to that of other EGFR TKIs in that it involves amplification of MET, PIK3CA, and NRAS. Since amivantamab targets MET, this could be a mechanistic explanation for its activity following mobocertinib.

As of now, the PAPILLON trial has defined amivantamab in addition to chemotherapy as the standard of care for first-line palliative therapy. Full publication of the EXCLAIM 2 trial that investigated first-line mobocertinib is still awaited.

In countries where amivantamab is not available as a first-line treatment, sequencing therapy following platinum might still be an issue. The real-world PFS of patients treated with amivantamab in the second- or a later-line therapy has been estimated to be 5.2 months (95% CI, 4.2–NE), which seems to be in a similar range as the results described above [20]. In the absence of comparative trials, we believe that the sequence of EGFR Ex20-specific therapies is determined by tolerability (discontinuation rate, 4% versus 17% in the CHRYSALIS and EXCLAIM/MOON studies, respectively), patient preference, and, unfortunately, drug availability and reimbursement.

## 4. Material and Methods

### 4.1. Study Design

We retrospectively analyzed patients with advanced EGFR Ex20 mutation-positive NSCLC treated with mobocertinib in an early access program between July 2020 and April 2023 (data cutoff date: 5 April 2023). Patients were treated at 34 centers in 11 European countries (Austria, Belgium, Croatia, Germany, Finland, France, Slovenia, Sweden, Switzerland, The Netherlands, and the United Kingdom), Israel, and Australia. The majority of patients were recruited from Austria and the National Network Genomic Medicine Germany (nNGM). Patients with active brain metastases were eligible for inclusion. The only exclusion criterion was missing informed consent, if required by law. The study was carried out in accordance with the Code of Ethics of the World Medical Association (Declaration of Helsinki).

### 4.2. Analysis of Clinical Endpoints

Evaluation of response was performed at the individual centers according to the Response Evaluation Criteria in Solid Tumors, version 1.1 (RECIST) and without a central review. For the objective response rate (ORR), patients with “not evaluable” as the best response to therapy were defined as nonresponders to provide a conservative ORR estimate, in case toxicity might have led to delay or omission imaging. The 95% confidence intervals for ORRs were calculated following the Clopper–Pearson method.

For PFS, the Kaplan–Meier curve was calculated from the start of mobocertinib treatment until the radiographic detection of disease progression, the start of a new line of antineoplastic therapy, or death, whatever occurred first; data were censored in the case where none of these events occurred by the last follow-up. For the duration of response (DOR), the Kaplan–Meier curve was calculated only for patients achieving a partial (PR) or complete response (CR) from the date of first detection of a PR or CR to the same event defined for PFS. Censoring was handled accordingly. For overall survival (OS), the Kaplan–Meier curve was calculated from the start of mobocertinib treatment until death, and data were censored in the case where none of these events occurred by the last follow-up. The median follow-up was calculated using the inverse Kaplan–Meier method. For each time-to-event endpoint, 95% confidence intervals were calculated. Cox regression was used for exploratory analysis of the covariates for PFS. EGFR mutation status was reported based on local clinical routine assay without central review. Assays were EGFR hotspot PCR (n = 11–13%), NGS panel sequencing (n = 72–85%), or unknown (n = 3–3%). TP53 mutation status was reported, if it was covered by local NGS assay. Further information on co-mutations was not available due to the heterogeneity of assays used. EGFR Ex20 subtypes—αC-helix (including most importantly p.A763insFQEA), near-loop (p.A767–p.P772), and far-loop (p.H773–p.C775)—were defined following the proposition of Robichaux et al. [21].

The frequency, type, and severity of treatment-related adverse events (TRAEs) were assessed by the treating physicians and according to the National Cancer Institutes’ Common Terminology Criteria for Adverse Events (CTCAE), version 5. An exploratory analysis of covariates for treatment discontinuation due to TRAEs was carried out using cross-tabulation and the chi-square test with Yates’s correction for continuity.

Statistical analyses were performed with IBM SPSS Statistics software, version 29.0. Figures were generated using GraphPad Prism 9 and Microsoft Office 2016.

### 4.3. Analysis of Postprogression Tumor Biopsies

All patients were assessed for available tumor material that was obtained during routine clinical examinations following disease progression on mobocertinib therapy and before initiation of further antineoplastic therapy. Samples were only processed if informed consent had been provided. Pre- and posttreatment tissue biopsies were obtained from five patients. In a sixth patient, a posttreatment liquid biopsy was obtained and compared to a pretreatment tissue biopsy. A board-certified pathologist analyzed tissue biopsies for morphologic transformation. Biopsy pairs were also analyzed by fluorescence in situ hybridization (FISH) for MET and HER2 amplification using the Zytolight Spec MET/CEN7 Dual Color Probe (ZytoVision) and PathVysion HER-2 DNA Probe Kit II (Abbott), respectively. Massive parallel panel sequencing (NGS) of DNA and RNA (optimized for fusions) was carried out following microdissection using the protocols “QIAseq Targeted DNA Panel, May 2017” (Qiagen) and “QIAseq Targeted RNA Scan Panel, August 2016” (Qiagen), respectively. Paired-end sequencing was carried out on the MiSeq platform (Illumina^®^). Bioinformatic work-up was performed using Genomics Workbench (CLC) and the following key filter parameters: coverage ≥200, allelic frequency ≥5% (≥1% in hotspots), exclusion of known polymorphisms and benign variants. HG19 and HG38 were used as reference genomes for DNA and RNA analysis, respectively. Please refer to Methods S1 for target lists of the custom DNA and RNA panels. Liquid biopsy was performed using Guardant360^®^ (Guardant).

In NGS, copy number variations (CNVs) were detected using Genomics Workbench (CLC) and an algorithm following the propositions of Li et al. and Nui and Zhang [22,23]. The difference in depth of coverage was assessed in comparison to control tissue from human tonsils. The cutoff was set at a 2.5x-fold difference in depth of coverage. This approach was validated internally against FISH for MET, EGFR, and ERBB2 (HER2).

### 4.4. Ba/F3 Model and In Silico Modeling

#### 4.4.1. Ba/F3 Model

Human wild-type EGFR was cloned into the MIY retroviral vector expressing enhanced yellow fluorescent protein (eGFP) as described previously.24 EGFR mutations were introduced using the Q5 site-directed mutagenesis kit (New England Biolabs) and verified by Sanger sequencing (Eurofins). 

Phoenix E helper-virus free ecotropic packaging cells (a kind gift from G. Nolan, Stanford, USA), were maintained in DMEM (Gibco) supplemented with 10% fetal calf serum (FCS). Ba/F3 cells were obtained from the German Resource Centre for Biological Material (DSMZ). Ba/F3 cells were maintained in RPMI 1640 medium (Thermo Fischer Scientific) containing 10% FCS in the presence of murine IL-3 at 2 ng/mL (PrepoTech/Thermo Fischer Scientific). For virus production Phoenix E cells were transduced using Lipofectamine 2000 (Thermo Fischer Scientific) according to manufacturer’s instructions. 1–2 × 10^5^ Ba/F3 cells were transduced by one round of spin infection (1200 g, 32 °C, 90 min). Retroviral supernatant was supplemented with 2 ng/mL IL-3, 4 μg/mL polybrene (Sigma-Aldrich), and 20% RPMI 1640 with FCS. 3 to 4 days after spin infection, cells were washed free of IL-3 and plated at a density of 0.25 × 10^6^ per mL in 2 mL medium per well in a 12-well plate and were cultured until fully transformed and afterwards maintained in IL3-free RPMI 160 medium. 

For inhibitor analysis, IL3-independent Ba/F3 cell lines expressing mutant EGFR were plated into 96-well plates (1.5 × 10^4^ per well), and inhibitors were added as indicated in triplicates. Cell growth was measured at 48 h using the WST-1 Proliferation Assay (Roche) according to the manufacturer’s instructions (Average in IC50 of at least 2 independent experiments is provided).

Erlotinib, gefitinib, afatinib, osimertinib, poziotinib and mobocertinib were purchased from Selleckchem (Houston, TX, USA). All inhibitors were dissolved in dimethyl sulfoxide (DMSO) to prepare stock solutions of 5 mM and were stored at –80 °C. The retroviral vector MIY hEGFR was a kind gift from Rama Krishna Kancha, Hyderabad, India and N. v. Bubnoff, Lübeck, Germany.

#### 4.4.2. In Silico Modeling

Modeller program [24] was used to obtain a 3D-structural model of the asymmetric tyrosine kinase dimer of the S768_D770dup variant. Protein Data Bank (PDB) entries 1M17 and 2GS6 were used as templates. To resolve steric clashes in the interdomain interface, energy minimization using NAMD program was performed [25]. The secondary G721S mutation was introduced into the S768_D770dup variant using PyMOL. Initial pose of non-covalently bound mobocertinib was obtained with AutoDock-GPU and AutoDockTools packages [26,27], and was found to be equivalent to the crystal pose of mobocertinib in a covalent complex with EGFR from PDB entry 7T4I. Mobocertinib was parametrized in GAFF2 force field using AmberTools21 [28], atomic charges were calculated using Gaussian as implemented in the PyRED web-server [29]. Binding pose of adenosine triphosphate (ATP) (in complex with magnesium ion) was obtained from PDB entry 1HCK. ATP molecule was parametrized in OL3 force field in combination with Steinbrecher and Case phosphate oxygen van der Waals radii [30]. Parameters for ATP polyphosphates were taken from Meagher et al. [31]. The protein variants were parametrized in the FF19SB Amber force field [32]. Four models of EGFR variants and their complexes were prepared: S768_D770dup and S768_D770dup+G721S in complex with mobocertinib or ATP with magnesium ion, respectively. All models were protonated at physiological pH, solvated in a rectangular box with OPC water model [33] and counterions for charge neutralization. The finally prepared systems were subjected to classical all-atom molecular dynamics (MD) (including the energy minimization, heating, equilibration, and the free run) using Amber20 as previously described [34]. For each model, three independent 75 ns-long trajectories were calculated. Langevin thermostat for MD simulations was set to 373 K to improve conformational sampling while using energy-consuming computational resources efficiently. The binding free energies of mobocertinib and ATP towards EGFR variants were calculated using MM/GBSA method (molecular mechanics generalised Born surface area) employing a single trajectory approach and the Onufriev, Bashford & Case GBOBC (I) solvent model [35] with default parameters. Calculations were performed independently for each frame within the last 20 ns of each MD trajectory with a 0.1 ns step. Entropic contributions were not considered in our study due to the dimension size of the molecular systems and following an assumption that the two EGFR TK variants that differ in G721S only, will have similar entropies.

## 5. Limitations and Conclusions

The MOON trial is limited by its retrospective nature and thus its unmonitored data, its limited sample size, particularly in the subgroup and biomarker analyses, and the lack of a central radiology review. Nevertheless, the trial is less prone to selection bias in young and fit patients compared with the available prospective data from the EXCLAIM study.

We conclude that mobocertinib is currently a valuable treatment option for NSCLC patients with EGFR Ex20 mutations. The high incidence of TRAEs warrants careful patient selection and monitoring, as well as evaluation of alternative and individualized dosing regimens.

## Figures and Tables

**Table 1:** Patient and disease characteristics.

Demographics ^a^	All Patients (N = 86)	Treatment-Naïve Patients (N = 14)	Pretreated Patients (N = 72)
Age, years Median (range)	67 (25–88)	75 (50–88)	65 (25–86)
Sex, n (%)			
Male	27 (31)	5 (36)	22 (31)
Female	59 (69)	9 (64)	50 (69)
Smoking status, n (%)			
Never smoker	54 (63)	9 (64)	45 (63)
Former smoker	31 (36)	5 (36)	26 (36)
Current smoker	1 (1)	0 (0)	1 (1)
Pack years in smokers (n = 31), n (%)			
Smoker (<30 py)	25 (29)	4 (29)	21 (29)
Heavy smoker (≥30 py) ^b^	6 (7)	1 (7)	5 (7)
ECOG performance status ^c^, n (%)			
0	44 (51)	9 (64)	35 (49)
1	34 (40)	4 (29)	30 (42)
2	6 (7)	1 (7)	5 (7)
3	2 (2)	0 (0)	2 (3)
Brain metastasis at baseline (n = 85), n (%)	28 (33)	4 (29)	24 (34)
Palliative regimens prior to mobocertinib, n Median (range)	2 (0–8)	NA	2
Previous regimens ^d^, n (%)			
Platinum-based chemotherapy ^e^	29 (34)	NA	29 (40)
Nonplatinum-based chemotherapy ^f^	9 (10)	NA	9 (13)
Platinum-based chemotherapy and	22 (26)	NA	22 (31)
anti-PD-1/PD-L1 therapy ^g^			
Anti-PD-1/PD-L1 therapy	7 (8)	NA	7 (10)
EGFR inhibitor	18 (21)	NA	18 (25)
Exon 20-targeted therapy ^h^	10 (12)	NA	10 (14)
Other ^i^	2 (2)	NA	2 (3)
Histology, n (%)			
Adenocarcinoma	84 (98)	14 (100)	70 (97)
Adenosquamous carcinoma	2 (2)	0 (0)	2 (3)
PD-L1 expression TPS (n = 72), n (%)			
<1%	33 (46)	6 (43)	27 (47)
1–49%	29 (40)	4 (29)	25 (43)
≥50%	10 (14)	4 (29)	6 (10)
TP53 mutation (n = 55), n (%)	22 (26)	3 (33)	19 (41)
EGFR mutation subtype (n = 66)			
Near-loop	53 (80)	12 (92)	40 (77)
Far-loop	10 (15)	0 (0)	10 (19)
Other (αC helix)	3 (5)	1 (8)	2 (4)

^a^ Percentages may not add to 100 because of rounding. ^b^ As defined by the National Lung Screening Trial [9]. ^c^ ECOG (Eastern Cooperative Oncology Group) performance status, with higher numbers indicating worse daily living capability. ^d^ Previous regimens defined as at least one dose of chemotherapy and/or immunotherapy or one dose of TKI therapy. ^e^ Five patients received bevacizumab with platin chemotherapy and pemetrexed. ^f^ Two patients received docetaxel with ramucirumab or nintedanib. ^g^ Eleven patients received atezolizumab and bevacizumab with platin chemotherapy and (nab)paclitaxel or pemetrexed. ^h^ Five patients received amivantamab, one patient received amivantamab in combination with lazertinib, and three patients received poziotinib. ^i^ One patient received trastuzumab emtansine, and one patient underwent transarterial chemoembolization of lung metastases. NA, not applicable; py, pack years; TKIs, tyrosine kinase inhibitors; TPS, tumor proportion score.

**Table 2:** Efficacy of mobocertinib.

	All Patients N = 86	Treatment-Naïve Patients N = 14	Pretreated Patients N = 72
Objective response rate, % (95% CI)	33.7 (23.9–44.7)	28.6 (8.4– 8.1)	34.7 (23.9–46.9)
Best response, n (%)			
Complete response	1 (1.2)	1 (7.1)	0 (0)
Partial response	28 (32.6)	3 (21.4)	25 (34.7)
Stable disease	33 (38.4)	6 (42.9)	27 (37.5)
Progressive disease	11 (12.8)	0 (0)	11 (15.3)
Not evaluable	13 (15.1)	4 (28.6)	9 (12.5)
Duration of response			
Median, months (95% CI)	8 (3.7–12.3)	14 (NE–NE)	6 (2.2–9.8)
Events/total, n/N (%)	18/29 (62)	2/4 (50)	16/25 (64)
Progression-free survival			
Median, months (95% CI)	5 (3.5–6.5)	6 (4.7–7.3)	5 (3.3–6.7)
Events/total, n/N (%)	67/86 (80)	10/14 (71)	57/72 (79)
Overall survival			
Median, months (95% CI)	12 (6.8–17.2)	NE	12 (7.1–16.9)
Events/total, n/N (%)	47/86 (55)	5/14 (36)	42/72 (58)
Median follow-up, months	21 (17.7–24.3)	12 (0.0–21.0)	22 (17.0–27.0)

Percentages may not add up to 100 because of rounding. CI, confidence interval.

**Table 3:** Treatment-related adverse events (TRAEs).

Patients (n = 86), n (%)									
TRAEs	Grade 1	Grade 2	Grade 3	Grade 4	Grade 5	Any Grade	Interruption	Dose Reduction	Discontinuation
Any event	71 (83)	57 (66)	29 (34)	2 (2)	2 (2)	82 (95)	36 (42)	44 (51)	15 (17)
Diarrhea ^a^	20 (23)	26 (30)	17 (20)	1 (1)	1 (1)	66 (77)	20 (23)	28 (33)	11 (13)
Acneifor rash	32 (37)	12 (14)	7 (8)	0 (0)	0 (0)	51 (59)	4 (5)	7 (8)	1 (1)
Dry skin	20 (8)	11 (12)	3 (3)	0 (0)	0 (0)	34 (40)	0 (0)	0 (0)	0 (0)
Nausea	13 (15)	13 (15)	3 (3)	1 (1)	0 (0)	30 (35)	4 (5)	5 (6)	4 (5)
Fatigue	15 (17)	10 (12)	4 (5)	0 (0)	0 (0)	30 (35)	0 (0)	0 (0)	0 (0)
Anorexia (decreased appetite)	14 (16)	13 (15)	2 (2)	0 (0)	0 (0)	29 (34)	1 (1)	5 (6)	1 (1)
Paronychia	15 (17)	12 (14)	0 (0)	0 (0)	0 (0)	27 (31)	2 (2)	2 (2)	0 (0)
Weight loss	18 (21)	1 (3)	0 (0)	0 (0)	0 (0)	21 (24)	0 (0)	1 (1)	0 (0)
Pruritus	14 (16)	5 (6)	2 (2)	0 (0)	0 (0)	21 (24)	0 (0)	1 (1)	0 (0)
Elevated creatinine	10 (12)	8 (9)	1 (1)	0 (0)	0 (0)	19 (22)	4 (5)	3 (3)	0 (0)
Oral mucositis	7 (8)	7 (8)	4 (5)	1 (1)	0 (0)	19 (22)	3 (3)	5 (6)	3 (3)
Vomiting	10 (12)	5 (6)	3 (3)	1 (1)	0 (0)	19 (22)	4 (5)	5 (6)	5 (6)
Anemia	11 (13)	3 (3)	0 (0)	0 (0)	0 (0)	14 (16)	0 (0)	0 (0)	0 (0)
Elevated ALT (SGPT) and/or AST	8 (9)	1 (1)	2 (2)	0 (0)	0 (0)	12 (14)	1 (1)	1 (1)	1 (1)
Skin pain	4 (5)	5 (6)	0 (0)	0 (0)	0 (0)	11 (13)	0 (0)	0 (0)	0 (0)
Elevated lipase	2 (2)	1 (1)	2 (2)	0 (0)	0 (0)	5 (6)	0 (0)	0 (0)	0 (0)
Rhinorrhea	4 (5)	0 (0)	0 (0)	0 (0)	0 (0)	4 (5)	0 (0)	0 (0)	0 (0)
Other adverse events ^b^	14 (16)	5 (6)	5 (6)	0 (0)	1 (1)	26 (30)	5 (6)	10 (12)	4 (5)

Grade of diarrhea, fatigue, liver toxicity, and tachycardia (other) was unknown for one patient each. Grade of skin pain and weight loss was unknown for two patients. In eight patients, lipase was not routinely measured. ^a^ One patient died due to grade 5 diarrhea in combination with grade 4 vomiting. ^b^ One patient died due to liver toxicity in combination with progression of liver and brain metastases.

## Data Availability

The raw data supporting the conclusions of this article will be made available by the authors on reasonable request within statutory and ethical regulations.

## Supplementary Materials

The following supporting information can be downloaded at: https://www.mdpi.com/article/10.3390/ijms25073992/s1.
