# Supplementary material for: Mobocertinib in Patients with EGFR Exon 20 Insertion-Positive Non-Small Cell Lung Cancer (MOON): An International Real-World Safety and Efficacy Analysis
Source: Int J Mol Sci. 2024 Apr 3;25(7):3992. doi: 10.3390/ijms25073992 (PMC11012872; doi:10.3390/ijms25073992)
Supplement: Supplementary file 1 [file ijms-25-03992-s001.zip › ijms-2912861-supplementary.pdf]

Supplementary Material

Table S1: Additional Patient Characteristics
Table S2: Exploratory analysis of covariates for progression-free survival
Table S3: In vitro sensitivity of EGFR exon 20 mutation with or without EGFR p.G721S to EGFR inhibitors
Table S4: EGFR mutation subtypes
Figure S1: Amivantamab following progression to mobocertinib
Figure S2: Treatment related adverse events
Methods S1: Targets of custom NGS panels

Table S1: Additional Patient Characteristics

Demographics[a]	All patients (N=86)	Treatment-naïve patients (N=14)	Pretreated patients (N=72)
Age, years			
Range	25-88	50-88	25-86
< 65	38 (44)	3 (21)	35 (49)
⩾65	48 (56)	11 (79)	37 (51)
Race, n (%)			
Asian	1 (1)	0 (0)	1 (1)
Non-Asian	85 (99)	14 (100)	71 (99)
Stage at initial diagnosis, n (%)			
Stage I	2 (2)	1 (7)	1 (1)
Stage II	7 (8)	3 (21)	4 (6)
Stage III	11 (13)	2 (14)	9 (13)
Stage IIIa	5 (6)	1 (7)	4 (6)
Stage IIIb	4 (5)	1 (7)	3 (4)
Stage IIIc	2 (2)	0 (0)	2 (3)
Stage IV	64 (74)	8 (57)	56 (78)
Stage IVa	18 (21)	3 (21)	15 (21)
Stage IVb	48 (56)	5 (36)	43 (60)
Stage at mobocertinib initiation, n (%)			
Stage III	1 (1)	1 (7)	0
Stage IV	85 (99)	13 (93)	72
Location of metastasis at mobocertinib initiation, n (%)			
Bone	48 (56)	5 (36)	39 (54)

Lung	43 (50)	9 (64)	38 (53)
Pleura	29 (34)	1 (7)	25 (35)
Brain	25 (29)	4 (29)	23 (32)
Liver	20 (23)	2 (14)	19 (26)
Adrenal gland	11 (13)	1 (7)	10 (14)
Lymph nodes	14 (16)	1 (7)	13 (18)
Other	17 (20)	2 (14)	15 (21)
Site of metastasis, n (%)			
0- 1	29 (34)	8 (57)	21 (29)
2–3	40 (47)	4 (29)	36 (50)
>3	19 (22)	2 (14)	17 (24)
Brain metastasis at baseline, n (%)			
Asymptomatic	16 (19)	3 (21)	13 (18)
Symptomatic	7 (8)	1 (7)	6 (8)
Unknown	5 (6)	0 (0)	5 (7)
Previous regimens curative setting, n (%)			
Neoadjuvant	2 (2)	0 (0)	2 (3)
Adjuvant	11 (13)	3 (21)	8 (11)
Definitive Chemoradiotherapy with Immunotherapy	1 (1)	1 (7)	0 (0)
Radiotherapy, n (%)			
Prior to mobocertinib administration			
Patients without radiotherapy	59 (69)	10 (71)	49 (68)
Neoadjuvant or post-operative thoracic radiotherapy	4 (5)	0 (0)	4 (6)

Stereotactic radiotherapy of brain metastasis	10 (12)	3 (21)	7 (10)
Palliative radiotherapy of bone or soft-tissue metastasis	11 (13)	3 (21)	8 (11)
Stereotactic radiotherapy for oligo metastasis	2 (2)	0 (0)	2 (3)
Whole brain radiotherapy	3 (3)	0 (0)	3 (4)
Palliative thoracic radiotherapy with or without lymph nodes	3 (3)	1 (7)	2 (3)
During mobocertinib administration			
No radiotherapy	78 (91)	13 (93)	65 (90)
Stereotactic radiotherapy of brain metastasis	2 (2)	0 (0)	2 (3)
Palliative radiotherapy of bone or soft-tissue metastasis	4 (5)	1 (7)	3 (4)

Data cut-off date: 05-Apr-2023. [a]Percentage may not be 100 because of rounding.

Table S2: Exploratory analysis of covariates for progression-free survival

Variable	Availability of Data	Hazard Ratio (95% CI)	P-value for Cox regression	Proportional hazards assumption
TP53: mutation vs. wildtype	N = 55 / 86 (64%)	0.959 (0.526 – 1.747)	0.891	True
Mobo given in firstline vs. later line	N = 86/86 (100%)	1.346 (0.685 -2.642)	0.389	Not true
Baseline brain metastases vs. no brain metastases	N = 86/86 (100%)	0.358 (0.209 – 0.615)	<0.001	True
ECOG 0 vs. ECOG 1-3	N = 86/86 (100%)	0.585 (0.358 – 0.956)	0.32	True
Female vs. male	N = 86/86 (100%)	0.977 (0.571 – 1.672)	0.934	True
Age ≤ 65 years vs. > 65 years	N = 86/86 (100%)	1.112 (0.685 – 1.805)	0.668	Not true
Discontinuation due to AE vs. no discontinuation	N = 86/86 (100%)	0.426 (0.231 – 0.787)	0.006	True
Near-loop vs. far-loop mutation	N = 65/86 (76%)	0.961 (0.404 – 2.285)	0.928	Not true

Hazard ratios and p-values were calculated using standard Cox regression without adjustment for multiple testing. The proportional hazards assumption was assessed visually on Kaplan-Meier plots. Significant results are labelled blue. Mobo = Mobocertinib. AE = Adverse event.

Table S3: In vitro sensitivity of EGFR exon 20 mutation with or without EGFR p.G721S to EGFR inhibitors

Mutation	Erlotinib	Gefitinib	Osimertinib	Afatinib	Poziotinib	Mobo-certinib
p.L858R	8,7	6,0	9,3	0,6	0,2	2,6
p.L858R + p.T790M	N.A.	N.A	34,2	98,4	26,6	32,8
p.D770_772dup	>3000	>3000	312,6	82,8	2,4	45,4
p.S768_D770dup	>3000	>3000	262,9	57,1	0,6	21,2
p.S768_D770dup + p.G721S	>3000	>3000	252,3	53,1	0,7	22,2

Ba/F3 cells retrovirally transduced with overexpression vectors encoding the EGFR exon 20 insertion p.S768_D770dup alone or in combination with the acquired EGFR p.G721S variant, and controls were deprived of IL-3 and treated with different concentrations of the indicated EGFR inhibitors. Half maximal inhibitory concentration (IC50) is provided in nM.

Table S4: EGFR mutation subtypes

Region	Mutation	Number of patients
αC helix	p.A763_Y764insFQEA	2
αC helix (but resistant to EGFRi)	p.Y764_V765insHH	1
Near-loop mutations	p.A767_S768insTLA	1
	p.A767_V769dup	15
	p.S768_D770dup	8
	p.V769_D770insQ	1
	p.V769_D770insTSV	1
	p.D770_N771insGF	1
	p.D770_N771insGL	1
	p.D770_N771insSVQ	1
	p.D770delinsGY	1
	p.D770_P772dup	2
	p.D770_N771insG	6
	p.N771dup	1
	p.N771_H773delinsLM	1
	p.N771_P772insT	1
	p.N771delinsGF	1
	p.N771delinsKH	2
	p.N771_H773dup	6
	p.P772_H773dup	1
	p.P772_H773insPNP	1
Far-loop mutations	p.H773dup	6
	p.H773_C775delinsPTP	1
	p.H773_V774delinsLM	1
	p.H773_V774dup	1
	p.H773_V774insPHPH	1

Amino acid changes as predicted from DNA variants based on transcript NM_005228.5 / ENST00000275493.7. Near-lopp/Far-loop classification is based on Robichaux et al..[9] Sequencing was performed by massive parallel sequencing panel analysis (NGS) and region-specific PCR assays in 87% and 13% of cases, respectively. Exact mutation name as depicted here was available for 65 of 86 patients.

Figure S1: Amivantamab following progression to mobocertinib

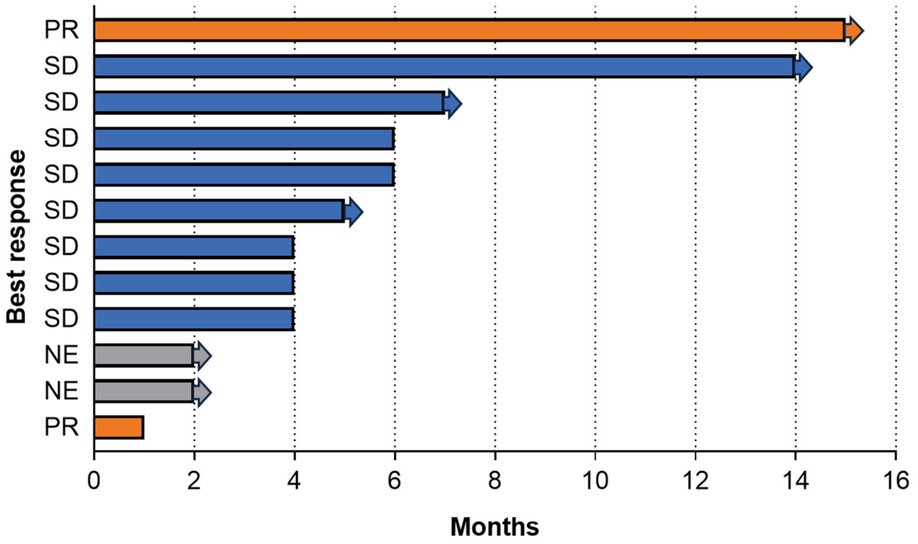

Swimmer plot for time on treatment. Arrows indicating ongoing treatment. Colors correspond to best objective response to amivantamab. PR: partial response (orange). SD: stable disease (blue). NE: not estimated (grey).

Figure S2: Treatment related adverse events

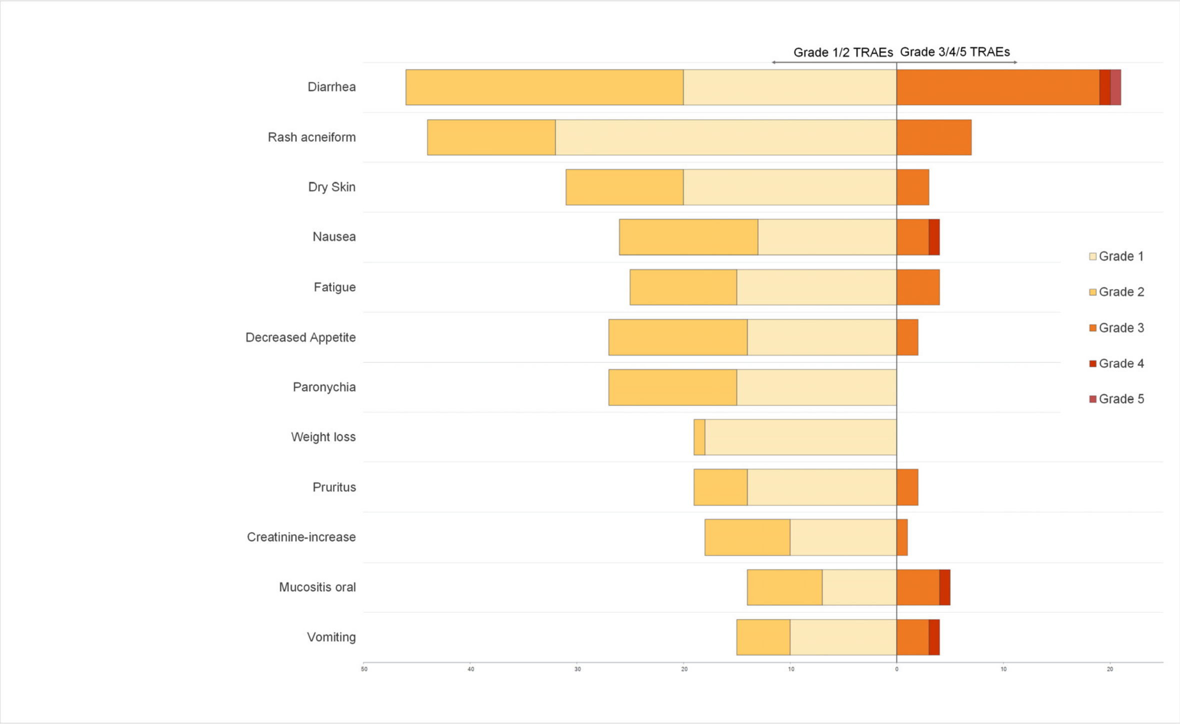

Data cut-off date: 05-Apr-2023. Treatment-related adverse events (TRAEs) that occurred at any grade in at least 20% of patients. The X-axis shows the number of patients who experienced the TRAE. The analysis included all patients who received at least one dose of mobocertinib. Relatedness of any adverse event to the treatment was assessed by the treating physician. TRAEs were graded as per Common Terminology Criteria for Adverse Events (CTCAE, version 5.0). Grading was not available for one case of diarrhea and fatigue and two cases of weight loss. Percentage may not equal to 100 because of rounding.

Methods S1: Targets of custom EGFR panels

	Gene	Transcript ID	Exons	Gene	Transcript ID	Exons
**nNGM Panel** Version 2.0	**ALK**	ENST00000389048.3	Exon 22-25	**BRAF**	ENST00000288602.6	Exon 11,15
	**CTNNB1**	ENST00000349496	Exon 3	**EGFR**	ENST00000275493.2	Exon 18-21
	**ERBB2**	ENST00000269571.5	Exon 8,19,20	**FGFR1**	ENST00000447712.2	Exon 4-7,10,12,13-15
	**FGFR2**	ENST00000358487.9	Exon 6-15,18	**FGFR2**	ENST00000457416	Exon 8, 9, 12, 18
	**FGFR3**	ENST00000440486.2	Exon 3,6,7,9,10,12,14,16,18	**FGFR4**	ENST00000292408.4	Exon 3,6,9,12,13,15,16
	**HRAS**	ENST00000311189.8	Exon 2-4	**IDH1**	ENST00000345146.2	Exon 4
	**IDH2**	ENST00000330062.3	Exon 4	**KEAP1**	ENST00000171111.10	Exon 2-6
	**KRAS**	ENST00000311936.7	Exon 2-4	**MAP2K1**	ENST00000307102.5	Exon 2,3
	**MET**	ENST00000397752.3	Intron 13,14 + Exon 14,16-19	**NRAS**	ENST00000369535.4	Exon 2-4
	**NTRK1**	ENST00000524377.5	Exon 13-17	**NTRK2**	ENST00000277120.7	Exon 14-19
	**NTRK3**	ENST00000360948.6	Exon 15-20	**PIK3CA**	ENST00000263967.3	Exon 8, 10,21
	**PTEN**	ENST00000371953.3	Exon 1-8	**RET**	ENST00000355710.8	Exon 10-18
	**ROS1**	ENST00000326873.11	Exon 34-41	**STK11**	ENST00000326873.11	Exon 1-9
	**TP53**	ENST00000269305.4	Exon 4-8			
**Mini-nNGM Panel** Version 2.0	**ALK**	ENST00000389048.3	Exon 22-25	**BRAF**	ENST00000288602.6	Exon 11,15
	**EGFR**	ENST00000275493.2	Exon 18-21	**KRAS**	ENST00000311936.7	Exon 2-4
	**MET**	ENST00000397752.3	Intron 13,14 + Exon 14,16-19	**NRAS**	ENST00000369535.4	Exon 2-4
	**PIK3CA**	ENST00000263967.3	Exon 8, 10,21			
**RNA Scan CFHS-10224Z-571 Panel** Whole Genes only	**ALK**	**BAG4**	**BRAF**	**CCDC6**	**CD74**	**CUX1**
	**DCBLD1**	**EGFR**	**EGFR-AS1**	**EML4**	**ETV6**	**EZR**
	**FGFR1**	**FGFR2**	**FGFR3**	**GOPC**	**HIP1**	**KIF5B**
	**KLC1**	**LOC107984219**	**LOC107985874**	**LRIG3**	**MET**	**MPRIP**
	**NRG1**	**NTRK1**	**NTRK2**	**NTRK3**	**NTRK3-AS1**	**RAD51**
	**RET**	**ROS1**	**SDC4**	**SLC34A2**	**STRN**	**TACC3**
	**TFG**	**TPM3**	**TPR**	**TRIM33**		
